# NO Exchange for a Water Molecule Favorably Changes Iontophoretic Release of Ruthenium Complexes to the Skin

**DOI:** 10.3390/molecules22010104

**Published:** 2017-01-08

**Authors:** Danielle C. A. S. de Santana, Karina Dias, Joel G. Souza, Abayomi T. Ogunjimi, Marina C. Souza, Roberto S. Silva, Renata F. V. Lopez

**Affiliations:** 1 School of Pharmaceutical Sciences of Ribeirão Preto, University of São Paulo, Ribeirão Preto 14040-903, SP, Brazil; danicristine@hotmail.com (D.C.A.S.S.); kadias@usp.br (K.D.); joelgsouza82@gmail.com (J.G.S.); togunjimi@usp.br (A.T.O.); marinacs@fcfrp.usp.br (M.C.S.); silva@usp.br (R.S.S.); 2 Department of Pharmaceutical Sciences, Federal University of Pernambuco, Recife 50670-901, PE, Brazil; 3 Department of Pharmaceutics, Faculty of Pharmacy, Obafemi Awolowo University, Ile-Ife 220282, Osun State, Nigeria

**Keywords:** ruthenium complex, iontophoresis, nitric oxide, in vitro skin penetration

## Abstract

Ruthenium (Ru) complexes have been studied as promising anticancer agents. Ru nitrosyl complex (Ru-NO) is one which acts as a pro-drug for the release of nitric oxide (NO). The Ru-aqueous complex formed by the exchange of NO for a water molecule after NO release could also possess therapeutic effects. This study evaluates the influence of iontophoresis on enhancing the skin penetration of Ru-NO and Ru-aqueous and assesses its applicability as a tool in treating diverse skin diseases. Passive and iontophoretic (0.5 mA·cm^−2^) skin permeation of the complexes were performed for 4 h. The amount of Ru and NO in the stratum corneum (*SC*), viable epidermis (*VE*), and receptor solution was quantified while the influence of iontophoresis and irradiation on NO release from Ru-NO complex was also evaluated. Iontophoresis increased the amount of Ru-NO and Ru-aqueous recovered from the receptor solution by 15 and 400 times, respectively, as compared to passive permeation. Iontophoresis produced a higher accumulation of Ru-aqueous in the skin layers as compared to Ru-NO. At least 50% of Ru-NO penetrated the *SC* was stable after 4 h. The presence of Ru-NO in this skin layer suggests that further controlled release of NO can be achieved by photo-stimulation after iontophoresis.

## 1. Introduction

The successes achieved with the discovery of cisplatin, a platinum complex extensively used as an antitumor compound, has boosted attempts by medicinal chemists to develop other compounds, such as ruthenium complexes, with fewer side effects and broader activity profile. The use of ruthenium complexes over the years span across diverse areas of science as they have been utilized as photochemical sensors [1], solar cells [2], organic reaction catalysts [3] and, particularly, as therapeutic agents for various diseases, such as cardiovascular disorders, HIV [4], malaria [5], and cancer [6].

The NAMI-complex (ImH)[*trans*-RuCl_4_(DMSO)(Im)] [7] and KP1019-[ImH][*trans*-RuCl_4_(Im)_2_] (im = imidazole) [8] are examples of the first anti-tumor metastasis inhibitors derived from ruthenium complexes [9] that were studied. Unfortunately, KP1019 did not progress to phase II trials, probably due to solubility issues preventing an increase of dosage above 600 mg per patient and the determination of a maximum tolerable dose [10] while NAMI-A’s combination with gemcitabine failed to produce improved result as compared to gemcitabine alone [11]. However, efforts can be directed at improving their drug selectivity, possibly enhancing these compounds activity at tumor site and reducing toxicity on healthy cells by application of external techniques, such as iontophoresis, that can direct active molecules to target sites [12].

One class of ruthenium compounds that have gained prominence over time are the ruthenium nitrosyl compounds [13], which may act as pro-drugs for the controlled release of nitric oxide (NO) in biological environments. NO is an important cell signaling molecule involved in many mammalian physiological processes [14], such as neurotransmission [15], blood pressure control [16], immune responses [17], cancer biology [18], and wound healing [19]. These ruthenium nitrosyl compounds, serving as NO donors can be activated by photo stimulation [20] and/or a redox reaction [21], allowing a site-specific release of NO for topical use. Moreover, the ruthenium complex formed after the NO release may also have antitumor properties, which could further act synergistically with the NO release. However, with respect to the skin penetration of these complexes, an important step for their effectiveness is compromised by their high molecular weight and presence of charges.

Iontophoresis is a physical method which involves the application of a constant low density electric current (≤0.5 mA/cm^2^) to facilitate drug delivery through the skin [22]. During iontophoresis, electric current passes through an electrolyte solution and the skin, carrying along ions present in the system. The passage of electric current through the electrolyte solution and the skin depends on the ions that make up the solution, as the ions facilitates the passage of electric current. Iontophoresis can increase the release of substances with short half-life directly into the tissues and allows better control of the drug transport, thereby ensuring an adequate dosage and good patient compliance [23].

**Figure 1:**
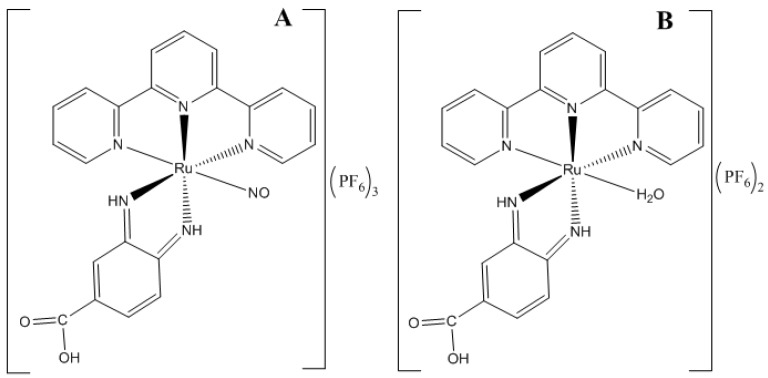
Chemical structure of complexes: (**A**) Ru-NO (MW = 949.9 g·mol^−1^) and (**B**) Ru-aqueous (MW = 792.4 g·mol^−1^).

**Figure 2:**
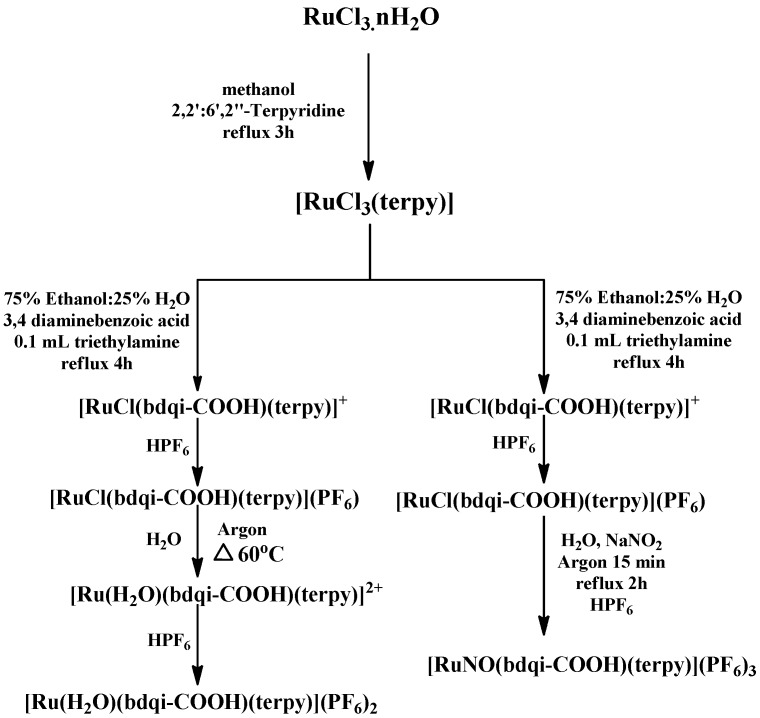
Synthesis pathway for obtaining complexes [Ru(bdqi-COOH)(terpy)(NO)](PF_6_)_3_, (Ru-NO) and [Ru(H_2_O)(bdqi-COOH)(terpy)](PF_6_)_2_, (Ru-aqueous).

In recent years, our group has explored the influence of iontophoresis on drug penetration into the skin and as a tool in treating skin tumors [24,25]. Two ruthenium complexes, namely Ru-NO ([Ru(bdqi-COOH)(terpy)(NO)](PF_6_)_3_, (bdqi = 1,2 benzoquinonediimine; terpy = terpyridine), a nitrosyl complex, and a Ru-NO complex modified by a simple exchange of the NO by a water molecule, [Ru(bdqi-COOH)(terpy)(H_2_O)](PF_6_)_2_, named Ru-aqueous in this study, were selected as model molecules for this study (Figure 1). The Ru-NO which has well-defined chemical characteristics [26] releases NO under reduction processes or light irradiation, producing the Ru-aqueous complex [27]. The Ru-aqueous complex, which is also a precursor of Ru-NO in the Ru-NO synthesis pathway (Figure 2) can be easily recovered by a simple precipitation process just before its reflux with sodium nitrite. Ru-aqueous possesses therapeutic activities which could be similar to the bioactivation of cisplatin within the cells, in which the substitution of chlorine molecules coordinated with platinum metal by water is indeed the active form of the cisplatin complex [28], an idea that could be applicable to the Ru-NO complexes. Therefore, the direct administration of the active metal complex, instead of the administration of its active precursor, at the site of the disease could promote a better control of the therapy. This way, iontophoresis may be an approach to improve subcellular localization and could be a useful tool to explain or enhance the biological activity of the metal-based compounds.

Thus, the objective of this study is to evaluate the influence of iontophoresis on the skin penetration of the model ruthenium complexes, Ru-NO and Ru-aqueous, with a view to assessing its applicability in the treatment of diverse skin diseases. Furthermore, as NO can be released from the Ru-NO complex into the different skin layers, the amount of NO released after iontophoresis was also quantified.

## 2. Results and Discussion

### 2.1. Ruthenium Complex Stability in Constant Low Intensity Electric Current

**Figure 3:**
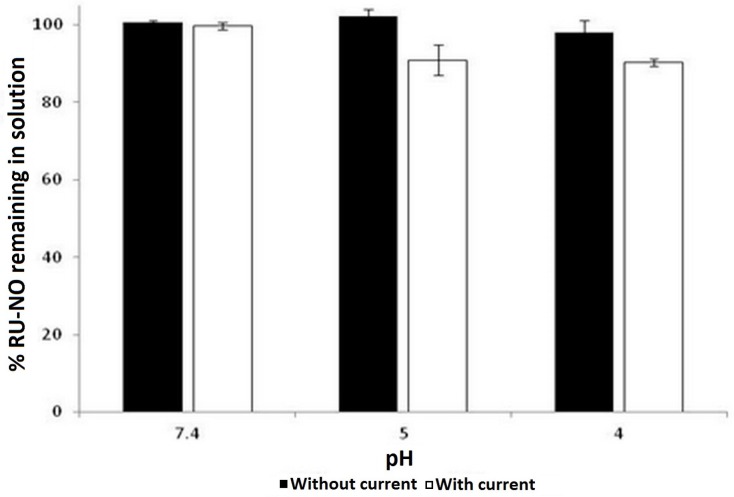
Stability of Ru-NO complex under application of 0.4 mA electric current for 6 h.

Figure 3 shows the stability of the Ru-NO complex under the application of a constant low intensity electrical current at different pHs for 6 h.

The result shows that the Ru-NO complex was stable in solution at the different pH studied for 6 h as there was no notable reduction in the initial Ru-NO concentration. However, the application of 0.4 mA electric current on Ru-NO complex solution led to a 10% reduction of Ru-NO complex at sub-physiological pH (pH 4 and 5) while there was no notable reduction in Ru-NO concentration at pH 7.4. This may be related to the equilibrium of nitrosyl ruthenium complexes as depicted in Equation (1). Ru-NO complex in solution exists in equilibrium depending on pH, as shown in Equation (1). As mentioned earlier, the dissolution of Ru-NO in water produced a solution of pH 4.5, which could be due to the utilization of the hydroxide ion from H_2_O.

[Ru^II^-NO^+^] + 2OH^−^ ↔ [Ru^II^-NO_2_^−^] + H_2_O (1)

Thus, to have a better control of the donor solution, passive and iontophoretic skin permeation studies were performed mostly at pH 4.5. In fact, NO release from Ru-NO solution at pH 4.5, upon light irradiation at specific wavelengths, has already been affirmed [29].

### 2.2. Irradiation of Ru-NO Complex

**Figure 4:**
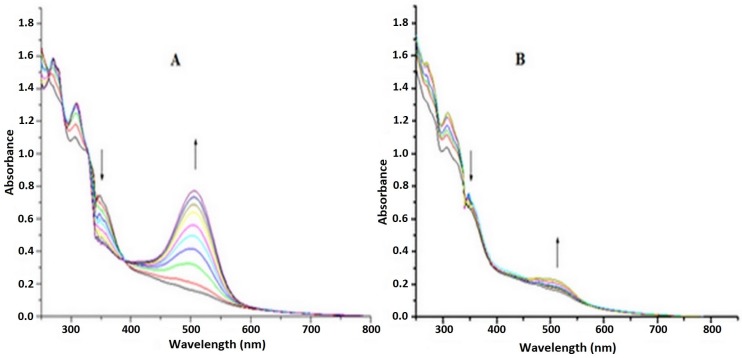
Spectral variation in Ru-NO complex in HEPES buffer (pH 7.4) after each 20 s laser irradiation at (**A**) 355 nm and (**B**) 532 nm.

Laser irradiation of Ru-NO solution at pH 7.4 was performed at two wavelengths of 355 nm and 532 nm, selected based on previously verified quantum yield [26]. Figure 4A shows the spectral variation in the Ru-NO complex in HEPES buffer (pH 7.4) after laser irradiation at 355 nm. The result shows a decrease in the band at 360 nm and an increase at 510 nm, indicating the release of NO, substitution of the NO by the H_2_O molecule, and the consequent formation of the Ru-aqueous complex which absorbed at 510 nm [26].

Irradiation at 532 nm, however, shows a minor spectral variation (Figure 4B). This small variation could be due to the low quantum yield of laser irradiation at 532 nm despite increasing its irradiation energy by 4.5 times.

**Figure 5:**
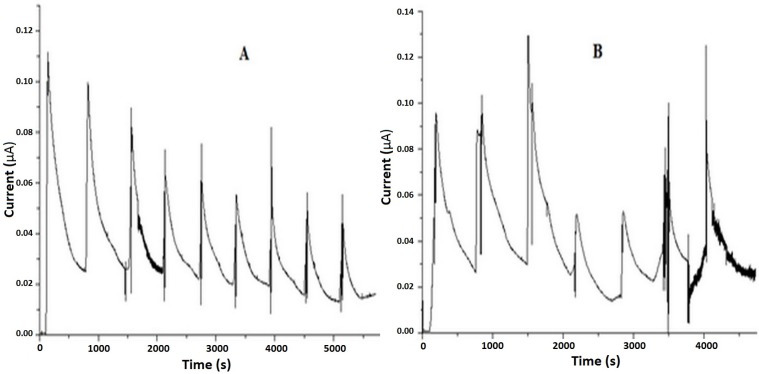
Qualitative cronoamperogram of NO release from Ru-NO complex in HEPES buffer (pH 7.4) laser irradiated at (**A**) 355 nm (laser power 10 mW) and (**B**) 532 nm (laser power 30 mW).

The qualitative result of the current-NO profile of Ru-NO complex in HEPES buffer (pH 7.4) registered by the NO meter after laser irradiation at 355 and 532 nm are as shown in Figure 5.

In situ NO monitoring during photo-irradiation processes has been a way of confirming photochemical release of NO in compounds [30]; a rapid increase in the signal indicates the release of NO when samples are under the photobeam and a decrease in the signal when the photobeam is interrupted, the decrease being accounted for by the utilization or consumption of NO mainly by oxidation [31]. Therefore, the release of NO from the Ru-NO can be activated by light at a specific wavelength in both acidic [29] and physiological pH (Figure 5).

### 2.3. In Vitro Skin Permeation

**Figure 6:**
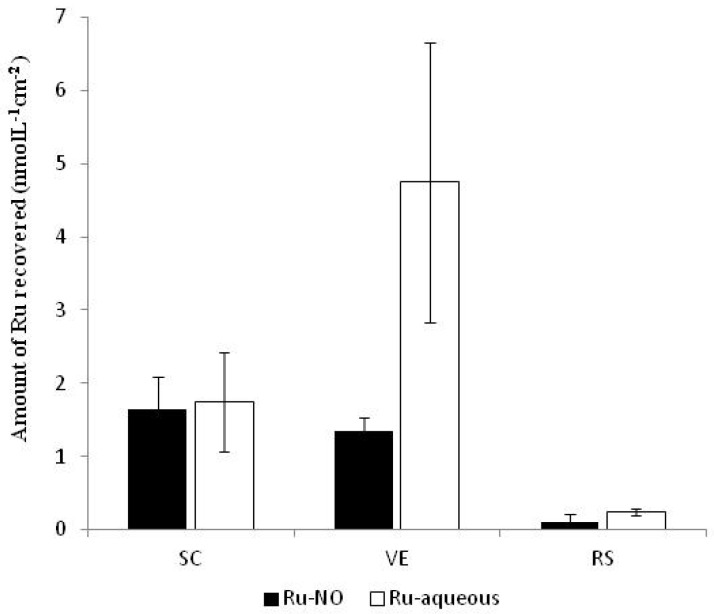
Amount of Ru recovered from the SC, viable epidermis and receptor solution after 4 h of passive permeation. Ru was converted to Ru-NO (MW = 949.9 g·mol^−1^) or Ru-aqueous (MW = 792.4 g·mol^−1^) depending on the donor complex added to the diffusion cell (mean ± SD, *n* = 5).

Figure 6 shows the cumulative amounts of Ru-NO and Ru-aqueous in the SC, viable epidermis, and receptor solution after 4 h of passive permeation. It should be noted that these experiments were analyzed by ICP-MS and, as such, only the Ru was determined. Thus, to analyze the results, all Ru found after the experiments with Ru-NO or Ru-aqueous were converted into Ru-NO or Ru-aqueous respectively.

Although, Ru-aqueous has the tendency to cross the SC more easily than the Ru-NO, probably due to Ru-aqueous’ smaller molecular weight, the result showed that Ru-NO penetration into the SC, viable epidermis, and receptor solution was statistically similar to that of Ru-aqueous (*p* > 0.05). Table 1 shows the influence of iontophoresis on transcutaneous penetration of the complexes.

**Figure 7:**
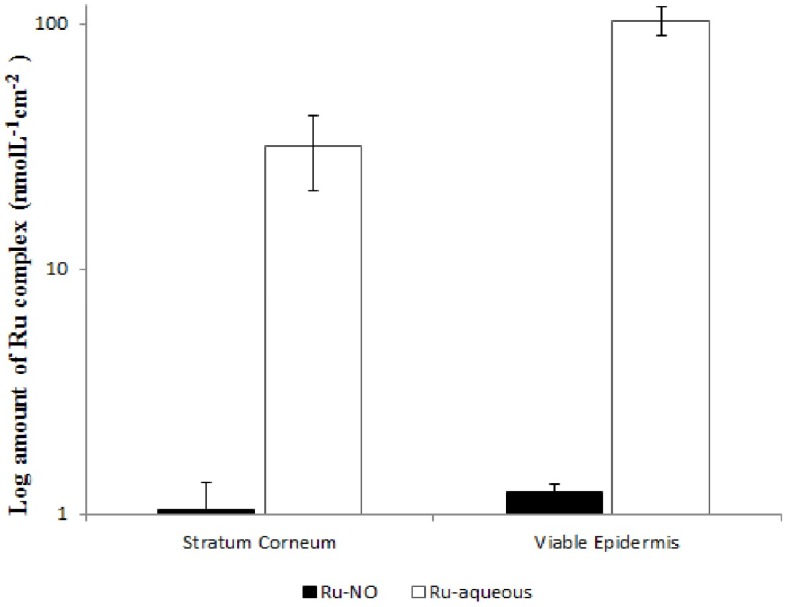
Ru-NO and Ru-aqueous recovered from the SC and viable epidermis after 4 h of iontophoresis.

As shown in Table 1, iontophoresis increased the permeation of Ru-NO by more than 15 times, while there was a more pronounced iontophoretic Ru-aqueous permeation of about 400 times when compared to the passive permeation of the two complexes, respectively. Iontophoresis also resulted in a more than an order of magnitude of higher accumulation of Ru-aqueous into the SC and viable epidermis when compared to the amount of Ru-NO recovered from the two skin layers (Figure 7).

Transport of substances through the skin under the application of iontophoresis occurs by the summation of the passive, electromigration, and electroosmotic flow. The electromigration flow ($J_{x}^{ER}$) can be described as an orderly movement of ions (*x*) towards the skin which depends on the electric current (*i_x_*) flow, skin area (*A*) involved in the transport, Faraday constant (96,500 C.mol^−1^), and charge of the ion as shown in Equation (2) [23].

(2) $$J_{x}^{ER}=\frac{1}{z_{x}AF}\;i_{x}$$

The pKa of the complexes, estimated using the MarvinSketch software (ChemAxon, Budapest, Hungary) was about 3.2. Although, the MarvinSketch software does have some limitations, particularly in relation to complex’s structure, the values calculated were in accordance with the expected pKa of benzoic acid which is about 4. Thus, both complexes would be cationic at the pH iontophoresis was performed, with the Ru-NO presenting a 2+ ion charge while Ru-aqueous presenting a 1+ ion charge. The higher ion charge of Ru-NO complex should produce higher iontophoretic flux than the Ru-aqueous [32]. However, as shown in Figure 7, Ru-aqueous permeated the skin much easier than Ru-NO upon application of iontophoresis. Therefore, it could be said that electroosmotic flow and not only electromigration certainly influenced the iontophoretic penetration of these high molecular weight complexes, particularly Ru-NO.

Electroosmotic flow, which is a convective solvent movement from the anode towards the cathode, decreases with the decreasing pH of solution in contact with the SC. Importantly, when the skin is in contact with solutions with pH values near 7, the carboxylate groups associated with amino acid residues present in the skin are ionized, favoring transport of cations and, consequently, the transport of water. At pH values close to 4, the carboxylate groups associated with the SC do not dissociate, thereby decreasing electroosmotic flow from the anode [33]. Thus, the iontophoretic permeation of Ru-NO at pH 4.5 might have been hindered by a decrease in the electroosmotic flow. However, Ru-aqueous solution might have suffered a contribution from electroosmosis at pH 5 [34].

**Figure 8:**
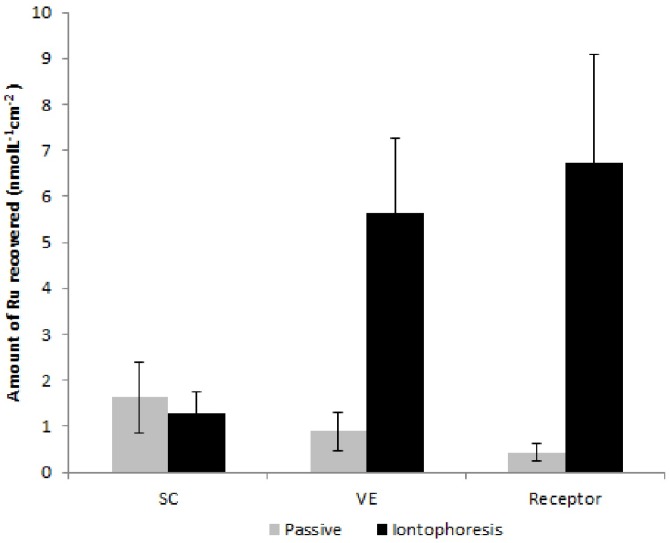
Amount of Ru recovered from the SC, VE, and receptor solution after 4 h of passive and iontophoretic permeation of Ru-NO dissolved in aqueous solution at pH 7.

To elucidate and improve the contribution of electroosmotic flow, iontophoretic delivery of a Ru-NO solution with pH adjusted to 7 was evaluated. Figure 8 shows the amount of Ru recovered from the skin layers and receptor solution after 4 h of passive and iontophoretic permeation using a Ru-NO solution adjusted to pH 7.

As expected, iontophoresis notably increased Ru penetration into the viable epidermis (six times) and receptor solution (16 times) when compared to the passive permeation. Although the amount of Ru-NO recovered from the receptor solution after iontophoresis at pH 7.4 was about five-fold higher than the amount recovered at pH 4.5 (Table 1), passive permeation of Ru-NO at pH 7 (Figure 8) was also higher than at pH 4.5 (Figure 6). The increase in passive permeation of Ru-NO at pH 7 as compared to pH 4.5 was also experienced with the iontophoretic permeation of Ru-NO i.e., the ratio of iontophoretic/passive permeation at pH 4.5 and pH 7 was about 15-fold, respectively. This implies that, at pH 7, Ru-NO does not dissociate as expected. In fact, at pH 7, as shown in Equation (1), there is a mixture of [Ru^II^-NO^+^] and [Ru^II^-NO_2_^−^], implying that some charges of the Ru-NO complex have been neutralized, facilitating its passive skin permeation, but decreasing the contribution of electromigration when iontophoresis was applied. However, the contribution of electroosmosis must have been higher at pH 7 than at pH 4.5, thereby compensating for the decrease in the electromigration contribution. Nevertheless, iontophoresis of Ru-aqueous (Table 1) was an order of magnitude higher than the iontophoresis of Ru-NO even at pH 7 (Figure 8). Thus, the exchange of the NO molecule for water did facilitate a higher iontophoretic flux for the ruthenium complex. Although, the spatial arrangement of the two ruthenium complexes, their interactions with the stratum corneum and the presence of water molecule itself and not only their charges may be a factor responsible for the differences in the complexes electro-transport, the exact mechanism responsible for this, however, still needs to be investigated.

### 2.4. NO Release in the Skin

In order to quantify and verify the release of NO from Ru-NO after 4 h of iontophoresis, the amount of nitrite found in the SC, viable epidermis, and receptor solution after the addition of nitrate reductase enzyme was determined spectrophotometrically at 540 nm. The total concentration of nitrite detected is proportional to the amount of NO present in the sample [35]. Table 2 shows the total amount of NO (nmol·L^−1^·cm^−2^) quantified in the different layers of the skin and receptor solution after 4 h iontophoresis application.

Results show that the amount of NO recovered from the SC after iontophoresis with the Ru-NO complex solution is approximately five times higher than that recovered after iontophoresis application to a control aqueous solution (133 mmol·L^−1^ NaCl, pH 4.5), suggesting that Ru-NO was able to penetrate the SC upon application of iontophoresis. However, statistical analysis showed no significant difference (*p* > 0.05) between NO amounts recovered from the viable epidermis and receptor solution after iontophoresis application to Ru-NO complex solution and the control aqueous solution. The presence of nitrous species in the viable epidermis of the samples may not be attributed to Ru-NO diffusion but to the presence of specific substances such as cysteine and tyrosine [36]. It could be suggested that Ru-NO complex undergoes a redox reaction in the subsequent layers after the SC due to the presence of natural reducing agents in the viable epidermis, consequently leading to the release of NO and formation of its aqueous complex.

The conversion of NO (MW = 30.01 g·mol^−1^) recovered from the SC (18 nmol·L^−1^·cm^−2^, Table 2) to Ru-NO shows that 0.6 nmol·L^−1^·cm^−2^ of Ru-NO penetrated the SC. However, the amount of Ru-NO obtained by converting the Ru quantified by the ICP/MS technique to Ru-NO was about 1 nmol·L^−1^·cm^−2^ (Figure 7), suggesting that almost half of the Ru-NO that penetrated the SC lost its NO prior to quantification after 4 h of iontophoresis. As NO is a gas with a half-life less than 30 s in biological environments [16,37], the reduction in the amount quantified in the SC may have occurred before the analysis through NO release due to the presence of natural reducing agents in the SC. However, at least 50% of Ru-NO penetrated and was stable in the SC after 4 h of experimentation.

The release of NO from Ru-NO can also normally be achieved by the application of a light stimulus [29] (Figure 5) suggesting that after Ru-NO iontophoretic permeation, the skin could be further subjected to light stimulation, thereby targeting the release of NO into the SC for the treatment of diseases, such as psoriasis [38]. Moreso, the treatment of diseases in the deeper layers of the skin, such as skin tumors, with NO can be possible since the complex seems to diffuse into the deeper layers of the skin, although the controlled release of NO in this scenario is not associated with light stimulus, but rather on the ability of specific skin components in reducing the complex. In this case, we can take full advantage of ruthenium’s potential by leveraging its ultimately higher skin penetration when administered in the form of Ru-aqueous, probably leading to better treatment outcomes for diseases in the deeper layers of the skin.

In view of these results, it can be suggested that ruthenium complexes can provide a broad framework for the design of new therapeutic agents with multiple potential applications due to the inherent multiple characteristics of the ruthenium metal center coupled with diverse ligands possessing biological significance.

Although Ru-NO is a high molecular weight compound, evidence of its molecules in the SC, and that of Ru-aqueous in other layers of the skin with the application of iontophoresis, is an advance in medicinal chemistry of metallic compounds. Thus, the use of nitrosyl ruthenium compounds as a pro-drug in the stratum corneum, its molecules reaching the deeper layers of the skin on the application of iontophoresis, and its interaction with the biological microenvironment existing in the skin through a substitution reaction, such as in Ru-aqueous, can give rise to a wide array of new therapeutically active molecules capable of reaching specific targets within the skin.

## 3. Materials and Methods

### 3.1. Materials

Ruthenium chloride (III), bdqi (1,2 benzoquinonediimine), 2,2′:6′,2′′-terpiridine, silver chloride (99.99%), platinum wire, silver wire (0.5 cm diameter) obtained from Sigma-Aldrich, St Louis, MO, USA; Hepes (4-(2-hydroxyethyl)-1-piperazineethanesulfonic acid) and methanol were obtained from J.T. Baker, Center Valley, PA, USA; NO detection full commercial kit (ADI-917-020) from Emzo Life Sciences, Farmingdale, NY, USA; ethanol was obtained from Vetec, Duque de Caxias, RJ, Brazil. All solutions used were prepared from a reverse osmosis water purification system. All other chemical reagents were of analytical grade.

### 3.2. Synthesis and Characterization of Complexes

The complexes Ru-NO and Ru-aqueous (Figure 1) were synthesized and characterized based on a synthesis pathway (Figure 2) previously described in the literature [26], with few modifications. Briefly, 0.050 g of *cis*-[RuCl(bdqi-COOH)](PF_6_) salt was dissolved in water (30 mL) under heating (60 °C) for 1 h. Then, 0.5 mL of HPF_6_ was added to the solution to obtain *cis*-[Ru(H_2_O)(bdqi-COOH)](PF_6_)_2_, which was filtered, washed with 5 mL cold ethanol and 20 mL ether to produce a yield of 75%. The *cis*-[RuNO(bdqi-COOH)](PF_6_)_3_ was synthesized by dissolving 0.038 g of *cis*-[RuCl(bdqi-COOH)](PF_6_) salt (0.069 mmol) in water (30 mL) under an argon atmosphere; 0.024 g of NaNO_2_ (0.34 mmol) was added and refluxed for 1 h. Two milliliters of HPF_6_ was then added to the solution under continuous stirring. The resulting orange precipitate was collected by filtration, washed with diethyl ether, and stored under vacuum in the dark. Typical yield for *cis*-[RuNO(bdqi-COOH)](PF_6_)_3_ was 87%. Anal. Calc. for C22H17N6O3P3F18Ru: C, 27.82; H, 1.89; N, 8.83. Found: C, 27.90; H, 1.99; N, 8.99%.

During the synthesis process, UV-VIS spectroscopy was deployed to monitor and confirm the formation of the Ru-NO complex (λ = 510 nm, log ε = 3.65) in the aqueous solution [39]. The Ru-aqueous complex had the same electronic characteristics as the Ru-NO complex, however, the intensity of its band at 500 nm was higher than that observed in the same region for the Ru-NO complex. The difference could be attributed to a decrease in electron density at the dπ (Ru^II^) → π* (bdqi-COOH) overlap, which resulted from the coordination of metal ion to the nitrosyl ligand [39].

The complexes thus produced were dissolved in water and thereafter analyzed using a Shimadzu High Performance Liquid Chromatography (Shimadzu HPLC, Kyoto, Japan) Instrument System (LC-10 AD, Kyoto, Japan) equipped with a binary solvent pump (LC 10-aT VP), SPD-10A VP UV-VIS detector, CTO-10AS VP column oven, and autosampler model SIL 10AD, as described in literature [27]. Data acquisition and analysis were performed using a Shimadzu Control Module (SLC-10A) coupled to a computer system with Shimadzu LC solution software. The mobile phase consists of phosphate buffer (0.01 mol·L^−1^, pH 7.0), trifluoroacetic acid and methanol (85:0.87:15, *v*/*v*). Reverse phase chromatographic separation was carried out using a Shim-pack CLC-ODS C18 column (250 mm × 4.6 mm i.d., 5 μm, Shimadzu, Kyoto, Japan) and a Shim-pack CLC-ODS C18 (10 mm × 4 mm i.d., 5 μm, Shimadzu, Kyoto, Japan) as a guard column. The flow rate was set at 1.0 mL·min^−1^, and an oven temperature at 37 °C, while the injection volume was 100 μL. These chromatographic conditions, which allowed the identification of Ru-No and Ru-aqueous complexes, had retention times of 4.3 and 6.1 min respectively.

### 3.3. Constant Low Intensity Electric Current: Ruthenium Complex Stability

Saturated solutions of Ru-NO complex in isotonized HEPES buffer (25 mM) was prepared at three different pH of 4, 5 and 7.4. The stability of Ru-NO solutions at these pHs was then evaluated under constant low intensity electric current connected in series using Ag/AgCl electrodes. The anode (Ag) was immersed in the Ru-NO solution while the cathode (AgCl) was immersed in isotonized HEPES buffer (25 mM, pH 7.4). The solutions were connected by a salt bridge and subjected to an electric current of 0.4 mA for 6 h. After 6 h, the amount of Ru-NO remaining in the solution was quantified by HPLC. The stability of the complex in the absence of electric current was also evaluated. Experiments were performed in duplicate at all pHs.

### 3.4. Ru-NO Complex Light Irradiation Studies

Light irradiation studies of Ru-NO complex dissolved in HEPES buffer (25 mM) at pH 7.4 containing 133 mmol·L^−1^ NaCl were conducted using a laser at wavelengths of 355 and 532 nm. A saturated solution of Ru-NO complex was irradiated at 4 °C in a quartz cuvette. At a wavelength of 355 nm, the Ru-NO solution was laser irradiated for 20 s, with each second equivalent to 10 pulses of 20 mJ and the irradiation process performed nine times totaling 36,000 J of energy. At a wavelength of 532 nm, the saturated Ru-NO solution was laser irradiated for 60 s, with each second equivalent to 10 pulses of 30 mJ. The procedure was repeated four times totaling a 162,000 J energy. After each irradiation session, samples were evaluated for spectroscopic variation in the UV-VIS region.

#### In Situ Determination of NO Release

In order to elucidate NO release profile during the laser irradiation, an electrode with a selective membrane for NO and capacity to detect NO by electric current variation was used. The electrode was immersed into the Ru-NO formulation container and the current variation was recorded by ami-NO software (Innovative instruments Inc., Lancaster, SC, USA) which built a characteristic NO release profile. The electric current variation recording was synchronized with the start of irradiation.

### 3.5. In Vitro Skin Penetration

Skin penetration studies were performed using skin obtained from pig’s ear. The pig ear was collected immediately after slaughter (Refrigerator Pontal Ltda, Pontal, SP, Brazil), dissected, dermatomed (~500 μm) with a dermatometer (TCM 3000—NOUVAG), and stored in aluminum foil at −80 °C for no more than 30 days before use.

#### 3.5.1. Skin Integrity Study

In order to ascertain the integrity of the stratum corneum (SC, the skin permeation barrier) electrical resistivity of the skins were measured with an arbitrary function generator (Agilent 33220, 200 MHz, SHF Communication Technologies AG, Womelsdorf, PA, USA) by using a Franz diffusion cell model mounted with the dermatomed pig ear skin. An Ag/AgCl electrode (In Vivo Metrics) inserted into the receptor compartment of the diffusion cell was connected to the alternating current signal generator at 100 mV (RMS) potential and 10 Hz frequency. The system was connected to a multimeter (ET Minipa 2053) which further connects to a second electrode inserted into the donor compartment of the diffusion cell. The electrical current passing through the mounted skin was measured using the connected multimeter and the total resistance was calculated according to Ohms law. The actual skin resistance was calculated by subtracting the resistance of the diffusion system (without mounted skin) from the total resistance. The permeation surface resistivity was then obtained by multiplying the actual skin resistance by the skin area available for permeation. Only skins having resistivity values >50 KΩ [40] were considered to be intact and used in the study.

#### 3.5.2. Skin Permeation Studies

The dermatomed skin (with SC facing upwards) was mounted in the diffusion cell separating the donor compartment from the receiver compartment. The receiver compartment was filled with the receptor solution consisting of HEPES buffer made isotonic with NaCl (pH 7.4) under 300 rpm magnetic stirring. Exactly 1 mL of aqueous solution of Ru-NO or Ru-aqueous complex (320 μmol·L^−1^) was added to the donor compartment (0.8 cm^2^ surface area) in addition to 133 mmol·L^−1^ of NaCl. The pH of the ruthenium complex solutions which was 4.5 and 5.5 for Ru-NO and Ru-aqueous was not adjusted, respectively, before the experiments. An experiment with the Ru-NO complex solution, however, with pH adjusted to 7 was also performed. The amount of Ru complexes in the SC, viable epidermis and receiver solution was quantified (Section 3.6) after 4 h of passive permeation experiments.

For the iontophoresis experiments, Ag/AgCl electrodes previously described [41] were inserted into the donor and receptor compartment of the diffusion cell. The electrode inserted in the donor compartment was connected to the anode, while that in the receiver compartment was connected to the cathode of the power supply APH 500DM model (Kepco Power Supply^®^, Flushing, NY, USA). A constant electric current of 0.5 mA/cm^2^ was then applied for a period of 4 h. The amount of Ru complexes in the SC, viable epidermis and the receiver solution was then quantified (Section 3.6). Control experiments were performed by adding 1 mL of aqueous NaCl (133 mmol·L^−1^) to the donor compartment without the complexes.

#### 3.5.3. Skin Retention Studies

After 4 h of permeation experiment (passive and iontophoretic), the skin was removed from the diffusion cell, clipped to a smooth surface with the exposed SC facing up, and swabbed dry with gauze. The exposed SC (0.8 cm^2^ surface area) in contact with the donor solution was subjected to a ‘tape stripping’ technique [42] using 15 strips of adhesive tape (Scotch Book Tape no. 845, 3M) to remove the SC while the complete removal of the SC was confirmed by the appearance of a ‘shining’ viable epidermis surface after application of the tape strips. The 15 strips (containing extracted SC) were placed in a falcon tube and exhaustively extracted with 5.0 mL of water and methanol (2:1, *v*/*v*) under mechanical shaking (AP 56, Phoenix) for 1 min; it was further subjected to a 15 min ultrasonic bath (Ultra Cleaner Unique ^®^ 1400) and filtered using a 0.45 μm syringe filter before quantification of the complexes as described in Section 3.6.

The remaining skin (viable epidermis) immediately after the ‘tape stripping’ step was cut into pieces, transferred into a falcon tube containing water and methanol (2:1, *v*/*v*) and ground with a tissue homogenizer (Turratec Tecnal TE-102) for 1 min. The dispersion was centrifuged at 986× *g* (Heraeus Megafuge 16R, Thermo Fisher Scientific, Waltham, MA, USA) for 10 min, the supernatant filtered (0.45 μm syringe filter) and the complexes quantified as described under Section 3.6.

### 3.6. Quantification of Ru-NO and Ru-Aqueous in Skin Penetration Experiments

#### 3.6.1. ICP-MS

Inductively-coupled plasma-mass spectrometry (ICP-MS) with an Elan DRC II (Perkin-Elmer, Norwalk, CT, USA) unit was deployed to quantify the amount of Ru in the receptor solution and skin samples using previously established methods [43]. Calibration plots were generated using Ru standards and the Ru amounts were converted into Ru-NO or Ru-aqueous considering that the molar mass of Ru (102 g·mol^−1^) corresponds to 10.74% and 12.87% of the total molar mass of Ru-NO (949.9 g·mol^−1^) and Ru-aqueous (792.4 g·mol^−1^) complexes, respectively.

#### 3.6.2. Total NO Release

The amount of NO present in the skin after Ru-NO complex in vitro permeation studies was determined using a NO detection assay kit (Enzo^®^ Life Sciences, Farmingdale, NY, USA). The NO detection kit is based on an enzymatic conversion of nitrate to nitrite in the presence of a nitrate reductase enzyme, following the Griess reaction, and confirmed by the formation of a colored azo product. All analytical tests were carried out painstakingly according to manufacturer's instruction (catalog number 917-020-ADI).

After 4 h of Ru-NO skin permeation with or without application of iontophoresis, aliquots from the samples extracted from the SC, viable epidermis and the receptor solution were incubated for 30 min in a greenhouse humidified incubator (95% O_2_, 5% CO_2_, 37 °C) using 96-well plates with the addition of enzyme co-factors (25 μL NADH, 50 mL Griess reagent I, 50 uL Griess reagent II) and nitrate reductase for the reduction of the nitric oxide present in the samples to nitrite. Absorbance was measured at 540 nm using an automatic microplate reader (Biorad EIA READER 2550) and the results obtained were evaluated by comparing with a standard curve of sodium nitrate (1.0 mmol·L^−1^), while data was expressed in μmol·cm^−2^ of NO.

### 3.7. Statistical Analysis

Statistical analysis of results obtained from the in vitro permeation study was performed using GraphPad Prism software (5.0, GraphPad, La Jolla, CA, USA). The results were expressed as mean ± SD and subjected to analysis of variance (ANOVA) and group comparison by Tukey’s post hoc test at 5% significance.

## 4. Conclusions

The study has shown that a change of the NO ligand to water significantly altered the iontophoretic permeation of ruthenium complexes. The passive permeation of Ru-NO and Ru-aqueous was quite similar; however, the application of iontophoresis for 4 h significantly increased the amount of ruthenium recovered from the skin and that which passed through it. A decrease in the pH of dissolved Ru-NO in solution, as compared to Ru-aqueous solution, could have contributed to the reduction in iontophoretic permeation of Ru-NO due to consequent decrease in electroosmotic flow. About 50% of Ru-NO that penetrated the SC did not lose its NO to unspecific reactions, suggesting that the further release of NO can be controlled by photo-stimulation after iontophoresis.

## Figures and Tables

**Table 1:** Ru-aqueous and Ru-NO amount quantified in the receptor solution after 4 h of passive or iontophoretic permeation with the same initial molar concentration (mean ± SD, *n* = 5).

Ru Complex	Passive (nmol·L^−1^·cm^−2^)	Iontophoresis (nmol·L^−1^·cm^−2^)
Ru-NO	0.1 ± 0.1	1.5 ± 0.2
Ru-aqueous	0.2 ± 0.0	91 ± 33

**Table 2:** Nitric oxide quantified in SC, viable epidermis (VE), and receptor solution (RS) after 4 h of iontophoresis application using 320 μmol·L^−1^ Ru-NO aqueous solution (pH 4.5).

Donor Solution	Total Concentration of NO * (nmol·L^−1^·cm^−2^)		
	SC	VE	RS
Control **	4 ± 1	15 ± 1	4 ± 3
Ru-NO	18 ± 0	18 ± 2	4 ± 3

* Determined by conversion to nitrite; ** Experiments conducted in the absence of Ru-NO compounds to determine the base concentration of NO in the skin. Mean ± SD, *n* = 5.
